# Analysis of multi-trait evolution across independently evolved cavefish populations reveals shared and independent evolution of suites of traits

**DOI:** 10.1098/rspb.2025.2719

**Published:** 2026-04-22

**Authors:** Stefan Choy, Maya Enriquez, Rianna Ambosie, Aubrey E. Manning, Briley Mullin, Roberto Rodriguez-Morales, Jennah Abdelaziz, Sarah Jacobson, Evan Lloyd, Naresh Padmanaban, Allison Kimmel, Solomia Lapko, Isabel Carino-Bazan, Helena Bilandžija, Alex C. Keene, Erik R. Duboue, Suzanne E. McGaugh, Johanna E. Kowalko

**Affiliations:** 1 Department of Biological Sciences, Lehigh University, Bethlehem, PA 18015, USA; 2 Department of Ecology, Evolution, and Behavior, University of Minnesota Twin Cities, Minneapolis, MN 55108, USA; 3 Department of Entomology and Nematology, University of California Davis, Davis, CA 95616, USA; 4 Department of Anatomy and Neurobiology, University of Puerto Rico, San Juan 00936, Puerto Rico; 5 Department of Biological Science, Florida State University, Tallahassee, FL 32304, USA; 6 Florida Atlantic University, Boca Raton, FL 33431, USA; 7 Florida Atlantic University Harriet L Wilkes Honors College, Jupiter, FL 33458, USA; 8 Division of Molecular Biology, Ruder Boskovic Institute Division of Molecular Biology, Zagreb 10000, Croatia; 9 Department of Biology, TAMU, College Station, TX 77843, USA

**Keywords:** cavefish, *Astyanax mexicanus*, repeated evolution, behaviour, evolution, genetics

## Abstract

Environmental perturbations often lead to the evolution of multiple traits. Determining whether shared genetic factors underlie multi-trait evolution is a central question in evolutionary biology. In the Mexican tetra, *Astyanax mexicanus,* cave-dwelling populations have repeatedly evolved multiple traits. The repeated evolution of these traits, paired with robust environmental differences between the surface and cave habitats, provide an opportunity to investigate the genetic basis of multi-trait evolution. Here, we investigate the extent to which shared genetic mechanisms underlie the repeated evolution of multiple traits in cavefish. Across cave populations, we find evidence for shared and distinct genetic mechanisms contributing to the evolution of individual traits. Further, multiple traits covary in cave–surface F2 hybrids. Moreover, many of the same pairs of traits covary in independently evolved cave populations. Finally, multiple traits differ between pigmented and albino F2 fish. Quantification of these traits in surface fish with mutations in the albinism gene *oculocutaneous albinism 2 (oca2*) reveals that mutations in *oca2* reduce bottom-dwelling behaviour in *A. mexicanus*. Together, these findings suggest that multitrait evolution occurs repeatedly through shared genetic factors across *A. mexicanus* cave populations. These results are consistent with pleiotropy and/or linkage playing a large role in multi-trait evolution in this species.

## Introduction

Populations inhabiting similar environments or subject to similar environmental pressures frequently evolve similar phenotypes. For example, in two species of pocket mice, *Chaetodipus intermedius* and *Perognathus flavescens*, and a species of fence lizard, *Scleporus undulatus*, divergent coloration has evolved between different populations that live on different coloured substrates such that individuals’ colours matches their substrate, reducing predation [1–4]. While the environmental pressures leading to repeated evolution have been studied extensively, the extent to which shared genetic mechanisms drive repeated evolution is less understood. Examples of both shared and distinct genetic mechanisms contributing to repeated evolution have been observed. For example, resistance to the cardiac glycosides in milkweed has independently evolved in at least three species, including the monarch butterfly, the red milkweed beetle, and oleander aphids, through mutations in the same gene encoding NA,K-ATPase [5,6]. Additionally, sticklebacks have repeatedly evolved reduced armour plating when colonizing different freshwater environments owing to selection on an *eda* allele that was present in the original saltwater populations [7]. By contrast, yellow pigmentation in *Drosophila novamexicana* and *D. americana* evolved via distinct genetic mechanisms [8]. While traits may evolve through a variety of mechanisms, more research is needed to understand why traits evolve through shared mechanisms in some instances, but independent mechanisms in others.

One factor that may influence how frequently a locus is utilized during evolution is pleiotropy. Historically, pleiotropic loci—loci that impact multiple traits—have been considered maladaptive in evolutionary contexts [9–11]. However, recent work suggests that pleiotropic loci are beneficial under certain conditions, such as when there is maladaptive gene flow or if a pleiotropic locus results in alterations to a suite of traits that together have positive fitness consequences [12–14]. Indeed, pleiotropic loci can drive the evolution of multiple traits. For example, when invading freshwater environments, stickleback populations evolved not only reduced plate armour, but also changes in body position when schooling, and changes in lateral line patterning through the pleiotropic *edar* gene [7,15–19]. Likewise, close linkage can promote multi-trait evolution in cases where independent genes produce adaptive changes in multiple traits [12,20]. For example, supergenes, or a group of genes inherited together that control multiple traits, have been drivers of rapid, multi-trait evolution in several different swallowtail butterfly species [21]. Thus, multiple traits may evolve through shared genetic loci repeatedly owing to the benefits of linkage and/or pleiotropy. Here, we directly test the extent to which repeated evolution of multiple traits occurs through the same genetic loci in the blind cavefish *Astyanax mexicanus*.

The freshwater fish *A. mexicanus* is a powerful system for investigating repeated evolution. *A. mexicanus* exists as two primary morphotypes: an eyed, riverine surface fish and a blind, subterranean cavefish. At least 35 distinct populations of cavefish have been identified, which derive from at least two colonization events from surface ancestors [22–25]. Cave populations have repeatedly evolved multiple behavioural, physiological and morphological trait changes relative to surface fish, including reduced sleep, reduced stress response behaviours, eye loss, albinism and increased numbers of superficial neuromasts (the sensory organs of the lateral line [26–33]. Additionally, cavefish remain interfertile with surface fish, enabling the generation of hybrids to investigate the genetic basis of specialized cave traits [34,35]. Together, these advantages make *A. mexicanus* a powerful model for investigating multi-trait evolution in response to the repeated colonization of a new environment.

Both genetic mapping studies and analysis of covariance of multiple cave-evolved traits in cave–surface hybrid fish suggest that concentrated genetic architecture may contribute to the evolution of cave traits in this species [36–43]. For example, quantitative trait loci (QTL) for different traits overlap in the *A. mexicanus* genome, and this occurs more frequently than expected by chance [36–39]. Moreover, functional studies demonstrate that the gene underlying albinism in *A. mexicanus* cave populations, *oculocutaneous albinism 2* (*oca2*), probably impacts at least four other cave-evolved traits: catecholamine levels, anaesthesia resistance, sleep and larval prey capture behaviour [42,44–47]. In addition, the gene *sonic hedgehog* has been implicated in the evolution of multiple cave traits, including eye degeneration, expansion of the jaw and tastebuds and alterations to the forebrain [48–50]. Together, these studies suggest that pleiotropy or linkage may be significant drivers of adaptation to cave life in *A. mexicanus* cavefish. However, most prior genetic studies have focused on only one cave population. Thus, whether evolution of multiple traits through shared genetic architecture has occurred repeatedly across *A. mexicanus* cave populations is currently unknown.

To systematically investigate the relationships between cave-derived traits, we established a pipeline to phenotype nine traits in surface fish, cavefish, and surface–cave and cave–cave hybrids. We assessed trait correlations in hybrid populations to determine whether there is evidence that multiple traits evolved through concentrated genetic architecture repeatedly across cavefish evolution. Furthermore, we investigated covariations between albinism and other traits directly by assessing bottom dwelling and eye size in *oca2* mutant surface fish. Together, this work suggests that shared genetic architecture between discrete traits may play an important part in the repeated evolution of the cave phenotypes in *A. mexicanus*.

## 2. Methods

### (a) Fish husbandry

Husbandry of *A. mexicanus* was carried out based on established methods [51,52]. Briefly, larvae were housed in incubators at 25°C on a 14 : 10 light : dark (L/D) schedule in glass bowls until 6 days post fertilization (dpf). Starting at 6 dpf, *A. mexicanus* larvae were moved to plastic tanks filled with 2 litres of water at a density of *n* = 30 per tank and were fed brine shrimp 1–2 times per day. Between 14 and 30 dpf, larvae were moved into 2.8 l plastic tanks on an Aquaneering recirculating water system. After 30 dpf, larvae were fed Gemma Micro 300 food (Skretting) once per day in addition to one to two brine shrimp feedings. Larvae were size-sorted every 2–4 weeks. After reaching approximately 3 cm in length, fish were transferred into 5-gallon tanks where they were fed 1 mm Ziegler pellets once to twice a day and kept at a maximum density of 15 fish per tank. Water temperature was maintained at 23 ± 1°C. Prior to behavioural assays, fish were fed only once per day for at least 2 weeks.

### (b) Hybrid generation

A nested hybrid population was generated by crossing individual *A. mexicanus* males from the Pachón, Molino and Tinaja cave populations to the same female surface fish. These surface and cave fish were bred and raised from a laboratory stock. This crossing structure generated surface–Pachón, surface–Molino and surface–Tinaja F1 hybrids that were all half-siblings. F2 hybrids were generated by incrossing F1 hybrids within each F1 cross (i.e. not between crosses), where all F2 hybrids share the same F0 surface fish ancestor. Separately, for phenotyping, a separate set of surface–cave F1 hybrids were generated from different parents. Cave–cave F1 hybrids were generated by crossing Pachón, Tinaja and Molino fish to one another. These crosses were done in both directions (e.g. PA♀ x MO♂ and MO♀ x PA♂). While cave–cave hybrids were generated bi-directionally, analysis revealed that the maternal/paternal origin of the parent did not influence the results. Therefore, the offspring from each set of bidirectional crosses were combined (i.e. Pachón × Molino and Molino × Pachón) in the analysis. For all comparisons with surface–cave or cave–cave F1 hybrids, pure surface or cave fish were assayed concurrently with a subset of hybrid fish. Notably, Pachón and Tinaja share a common ancestor more recently in time than either does with Molino and, therefore, we expect more complementation of traits in the wider crosses [25,53]. All fish in this study originated from laboratory stocks.

Our analyses of trait correlations in laboratory-derived F2 hybrids utilize relatively inbred lines; therefore, we expect the impact of standing variation and bidirectional gene flow to be largely muted by our experimental design. Trait correlations in F2 crosses depend on how alleles co-segregate, not on where those alleles evolved. However, if alleles affecting different traits are partially shared among populations, allele sharing would possibly reduce, obscure or increase the presence of correlations across populations.

### (c) Phenotyping pipeline

**Figure 1.**
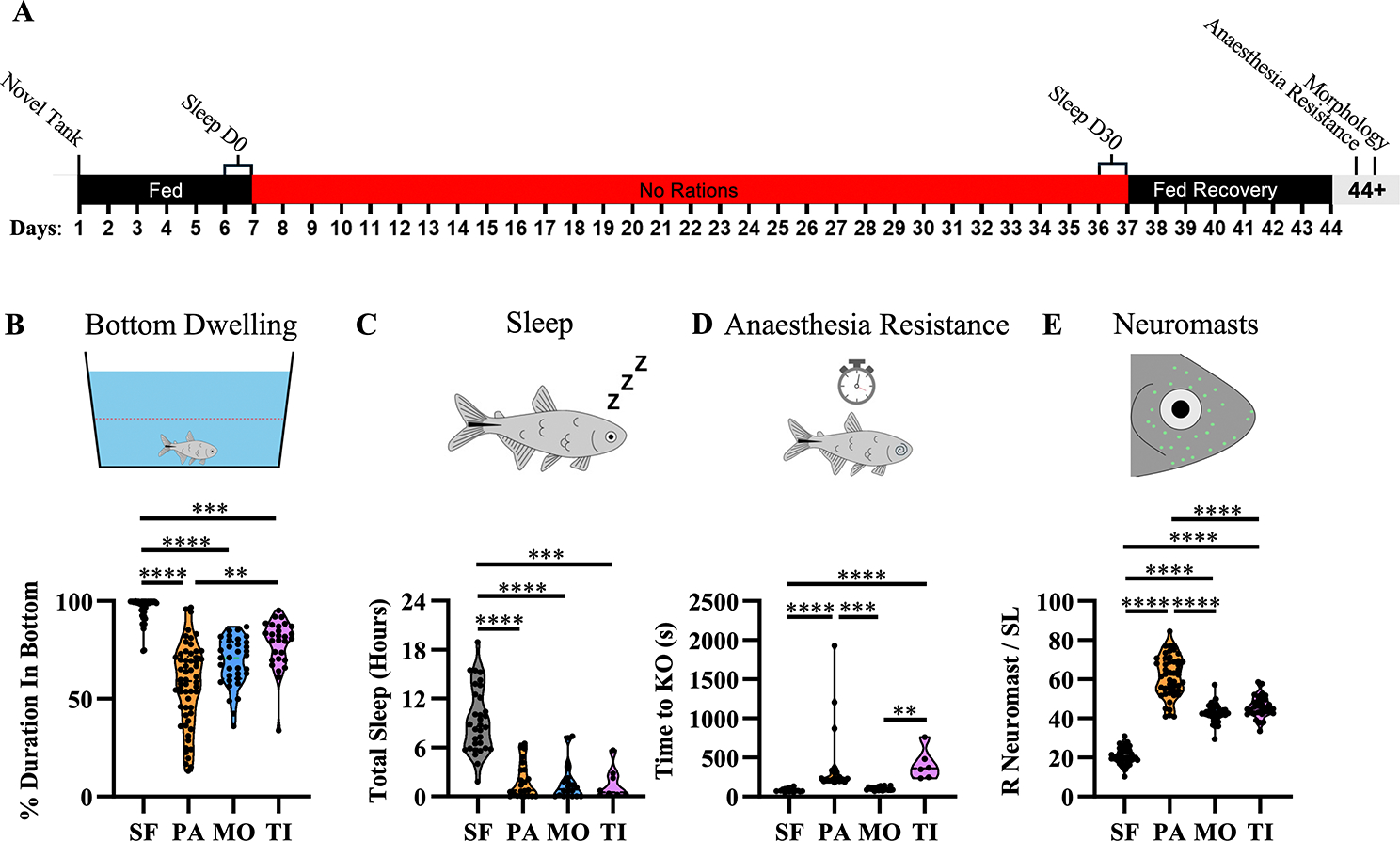
Cave populations of *A. mexicanus* repeatedly evolve traits that differ from surface populations. (A) Overview of the experimental pipeline that was used to assay adult surface fish, cavefish and hybrids. Eye morphology, neuromasts, presence/absence of pigmentation and standard length data were captured during the ‘Morphology’ section. (B) Percent of time spent bottom-dwelling during the novel tank assay (*N*: SF = 50, PA = 58, MO = 34, TI = 28; SF, PA, MO and TI represent surface fish, Pachón, Molino and Tinaja, respectively.). (C) Total sleep over 23.5 h during day 0 (D0) of the pipeline (*N*: SF = 32, PA = 23, MO = 20, TI = 8). (D) Time spent responsive following the addition of tricaine anaesthetic (*N*: SF = 14, PA = 20, MO = 19, TI = 6). KO=Knock out. (E) Number of superficial neuromasts located over the right suborbital 3 bone corrected by standard length (SL) (*N*: SF = 41, PA = 47, MO = 31, TI = 30). Right-side neuromasts over standard length were graphed, but statistical comparisons are of right-side neuromast residuals, calculated as described in the §2. Exact *p* values are reported in electronic supplementary material, table S1. Data points indicate values of phenotypes of individual fish. Levels of significance are represented with asterisks: *p* < 0.05 = *, *p* < 0.01 = **, *p* < 0.001 = ***, *p* < 0.0001 = ****.

Surface fish, Pachón, Molino and Tinaja cavefish, and hybrid fish were assayed for multiple behavioural, physiological and morphological traits (figure 1A). These traits were: stress response via a novel tank test to assess bottom-dwelling; sleep over 24 h; sleep over 24 h after 30 days of no rations; anaesthesia resistance; eye size; pupil size; cranial superficial neuromast number; and the presence or absence of pigmentation (figure 1). A subset of individuals went through a shortened pipeline where the 30 days of no ration period was omitted. These individuals do not have measurements for day 30 sleep but were assayed for anaesthesia resistance and morphological traits following day 0 sleep. For a separate project, fish were assayed for aggression via resident–intruder assays on the day following novel tank assays, and they were weighed prior to and following periods of no rations. Furthermore, while fish were recorded for 24 h to ascertain sleep phenotypes at days 0, 15 and 30 of no rations, only sleep data from the days 0 and 30 are reported here (Sleep D0 and Sleep D30 in figure 1A). Methods for phenotyping assays were based on published work [32,45,54,55]. Details on methods for phenotyping can be found in the electronic supplementary materials.

### (d) oca2 mutant surface fish

To directly test the role of pleiotropy in multi-trait evolution of *Astyanax* cavefish, we used a line of surface fish with a CRISPR-Cas9-induced 2-base pair (bp) deletion in exon 21 of *oca2* [42,56]. Here, fish heterozygous for the 2 bp deletion were incrossed, and offspring were raised to adult stages (>6 months). Albino and pigmented fish were raised separately to prevent competition; while cohabitation would be ideal, pigmented fish outcompete albino surface fish for food in lighted conditions [46], and in our experience, this results in differential growth rates and/or survival (Choy, 2021, unpublished data). All offspring from incrosses were assayed in the novel tank assay and then imaged using a Canon Rebel camera. After data collection, fish were anaesthetized and part of the tail fin was clipped for DNA extraction to genotype the pigmented individuals to distinguish heterozygous individuals from wild-type individuals [42,46,56] (see also, electronic supplementary materials).

### (e) Statistical analysis

Non-parametric tests (Mann–Whitney *U* tests, Kruskal–Wallis tests followed by Dunn’s multiple corrections, and Spearman’s rank correlation tests) were used for conservative estimates of statistical significance. A Hedge’s *G* test was used to calculate effect size for *oca2* mutant surface fish because the data had different sample sizes. Analyses were performed in Graphpad and R [57] and graphs were generated in Graphpad Prism (v. 10.3.0 for Windows, Graphpad Software, Boston, Massachusetts USA, graphpad.com). For additional details on statistical analysis and all statistics, see electronic supplementary material and table S1.

## 3. Results

### (a) Morphological and behavioural traits differ between surface fish and cavefish from multiple populations

To directly assess the extent to which morphological, behavioural, and physiological traits have repeatedly evolved across cavefish populations, we quantified and compared behavioural, physiological and morphological traits between surface fish and cavefish from the three cave populations, Pachón, Molino and Tinaja, (figure 1A). First, we assessed bottom-dwelling in response to a novel environment, which is a measure of stress response that was shown in previous studies to be significantly reduced in cavefish relative to surface fish [32]. Fish from all three cave populations spent significantly less time in the bottom half of the tank compared with surface fish (figure 1B). Pachón cavefish spent the least amount of time bottom-dwelling, spending significantly less time in the bottom half of the tank than Tinaja cavefish (figure 1B).

Next, we assessed baseline sleep, which is reduced in cavefish [31,58]. Sleep in *A. mexicanus* is defined by periods of inactivity longer than one minute [58]. In agreement with previous studies, we found that sleep is significantly reduced in fish from all cave populations compared with surface fish (figure 1C).

Cavefish have evolved a number of traits associated with resistance to starvation, including alterations to sleep in response to food deprivation [59]. To determine how lack of food impacts sleep in fish from different cave populations, fish from all populations were housed with no rations for 30 days. To assess the effects on sleep over this period, we recorded sleep prior to and immediately after the no rations period. Fish from all three cave populations show increases in sleep following a period of 30 days of no rations; however, only fish from the Tinaja and Molino populations had statistically significant increases in sleep compared with baseline sleep (electronic supplementary material, figure S1B–D). By contrast, surface fish did not show a significant directional change in sleep following 30 days of no rations compared with baseline sleep (electronic supplementary material, figure S1A). Similarly, in all populations, fish that were fed did not show differences in sleep after 30 days compared with baseline sleep levels, confirming that there was no effect of retesting (electronic supplementary material, figure S1). To directly compare food deprivation-induced changes in sleep between populations, we calculated the change in sleep for each individual starved fish. We found no statistically significant differences between populations in changes in sleep, although change in sleep was slightly increased in fish from the Molino and Tinaja populations (electronic supplementary material, figure S2F).

After at least seven days of recovery from no rations, anaesthesia resistance was assessed because previous work found that cavefish are more resistant to anaesthesia than surface fish [45]. When exposed to the anaesthetic tricaine, fish from all cave populations exhibited qualitatively increased anaesthesia resistance relative to surface fish, with Pachón and Tinaja cavefish taking significantly longer to succumb to anaesthesia than surface fish and Molino cavefish (figure 1D). Thus, our data demonstrate a shift in anaesthesia resistance consistent with past studies.

Analysis of morphological traits was consistent with previous studies. As reported previously for pigmentation [33,60], we observed reduced melanin pigmentation in Tinaja cavefish and albinism in fish from Pachón and Molino populations (data not shown). Cavefish initially develop eyes, which regress over the course of development [30,61]. Fish from all three cave populations have no external eyes or pupils as adults, whereas surface fish have large and fully formed eyes (electronic supplementary material, figure S2A–B). Cavefish also have expanded numbers of cranial superficial neuromasts compared with surface conspecifics [62–64]. We assessed the number of superficial neuromasts within the bounded area of the suborbital 3 bone. Fish from all three cave populations have significantly more superficial neuromasts than surface fish, and Pachón cavefish have significantly more neuromasts than Molino and Tinaja cavefish (figure 1E, electronic supplementary material, figure S2C–E). Together, these data demonstrate that while there are some differences in morphological, physiological and behavioural phenotypes between different cave populations, multiple traits have repeatedly evolved across cavefish populations.

### (b) Evaluation of modes of inheritance in different cave populations

**Figure 2.**
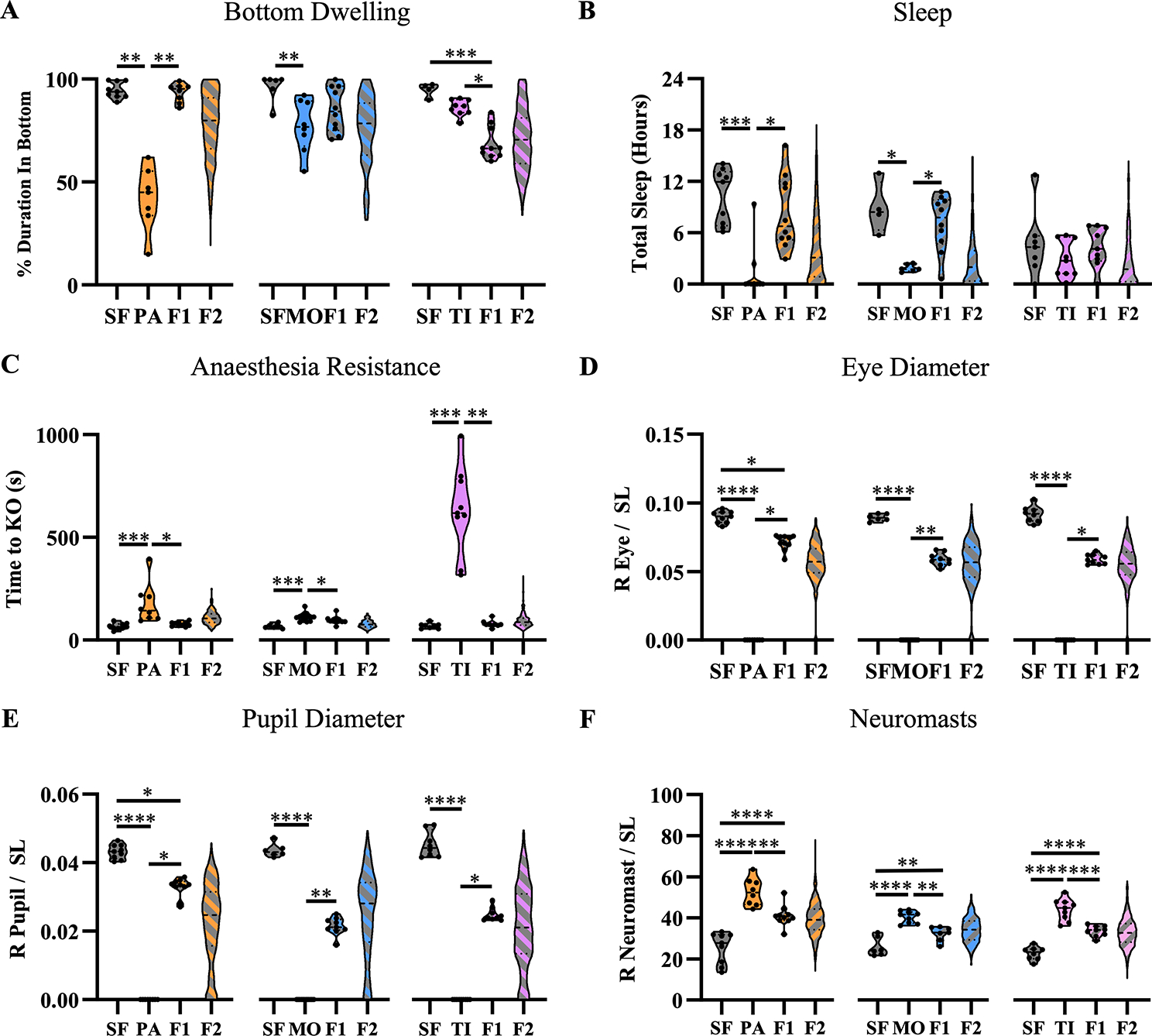
Surface–cave hybrids indicate heritability of cave-evolved traits. (A) Percent of time spent bottom dwelling during the novel tank assay (*N*: SF = 9, PA = 7, F1 = 8, F2 = 738; SF = 6, MO = 8, F1 = 10, F2 = 144; SF = 4, TI = 9, F1 = 10, F2 = 307). (B) Total sleep over 23.5 h during sleep day 0 (*N*: SF = 9, PA = 9, F1 = 10, F2 = 489; SF = 4, MO = 6, F1 = 10, F2 = 151; SF = 7, TI = 9, F1 = 9, F2 = 243). (C) Time spent responsive following the addition of tricaine anaesthetic (*N*: SF = 9, PA = 8, F1 = 10, F2 = 693; SF = 6, MO = 12, F1 = 10, F2 = 124; SF = 7, TI = 9, F1 = 9, F2 = 243). KO = Knock out. (D) Dorsal–ventral right eye diameter corrected by standard length (SL) (*N*: SF = 9, PA = 8, F1 = 10, F2 = 777; SF = 6, MO = 12, F1 = 9, F2 = 184; SF = 8, TI = 9, F1 = 9, F2 = 325). Note that while SF–MO and SF–TI comparisons are not statistically significant, F1 fish from both of these populations have smaller eyes relative to surface fish (SF–SFMOF1: SF median = 0.089, SFMOF1 median = 0.058, *p* value = 0.18; SF-SFTIFI: SF median = 0.092, SFTIF1 median = 0.058, *p* value = 0.0586). (E) Dorsal–ventral right pupil diameter corrected by standard length (*N*: SF = 9, PA = 8, F1 = 10, F2 = 775; SF = 6, MO = 12, F1 = 9, F2 = 184; SF = 8, TI = 9, F1 = 9, F2 = 326). Note that while SF–MO and SF–TI comparisons are not statistically significant, F1 fish from both of these populations have smaller eyes relative to surface fish (SF–SFMOF1: SF median = 0.043, SFMOF1 median = 0.021, *p* value = 0.1817; SF–SFTIFI: SF median = 0.044, SFTIF1 median = 0.024, *p* value = 0.0586). (F) Number of superficial neuromasts located over the right suborbital 3 bone corrected by standard length (*N*: SF = 9, PA = 8, F1 = 10, F2 = 772; SF = 6, MO = 12, F1 = 9, F2 = 181; SF = 8, TI = 9, F1 = 9, F2 = 319). Right-side neuromasts over standard length were graphed, but statistical comparisons are of right-side neuromast residuals, calculated as described in §2. Data points indicate values of phenotypes of individual fish. Data points are not shown for F2 hybrids. All statistical comparisons were performed between surface fish, cavefish and F1 hybrids. No statistical comparisons were performed with F2 hybrids. Exact *p* values are reported in electronic supplementary material, table S1. SF, PA, MO and TI represent surface fish, Pachón, Molino and Tinaja, respectively. Levels of significance are represented with asterisks: *p* < 0.05 = *, *p* < 0.01 = **, *p* < 0.001 = ***, *p* < 0.0001 = ****.

Next, we quantified traits that showed significant differences between surface and cave fish in cave–surface F1 and F2 hybrid fish to determine whether modes of inheritance for these traits are shared across cave populations. Similar patterns of inheritance were observed in cave–surface F1 hybrids across all populations for four traits: all F1 hybrids are intermediate between surface and cave phenotypes for eye size (figure 2D, electronic supplementary material, figure S3A), pupil size (figure 2E, electronic supplementary material, figure S3B) and neuromast number (figure 2F, electronic supplementary material, figure S3C–E). Furthermore, all F1 hybrids exhibit statistically significantly reduced anaesthesia resistance relative to their corresponding cavefish populations, suggesting a surface-like anaesthesia resistance for F1s (figure 2C). For other traits, however, quantification in surface–cave F1 hybrid fish from different cave populations suggests different inheritance patterns between cave populations. Surface–Pachón and surface–Molino F1 hybrids exhibit surface-like levels of bottom dwelling, while surface–Tinaja F1s exhibit reduced bottom dwelling relative to surface fish, which is a cave-like phenotype (figure 2A). Sleep in surface–Pachón F1 hybrids and surface–Molino F1 hybrids is surface-like, while surface–Tinaja F1s appear to be intermediate in phenotype and not significantly different from surface or Tinaja parentals (figure 2B). The variation in inheritance across traits in cave–surface F1 hybrids suggests that there is variation between cave populations in the underlying genetic basis of the traits we examined here.

### (c) Investigating traits in cave–cave hybrids

**Figure 3.**
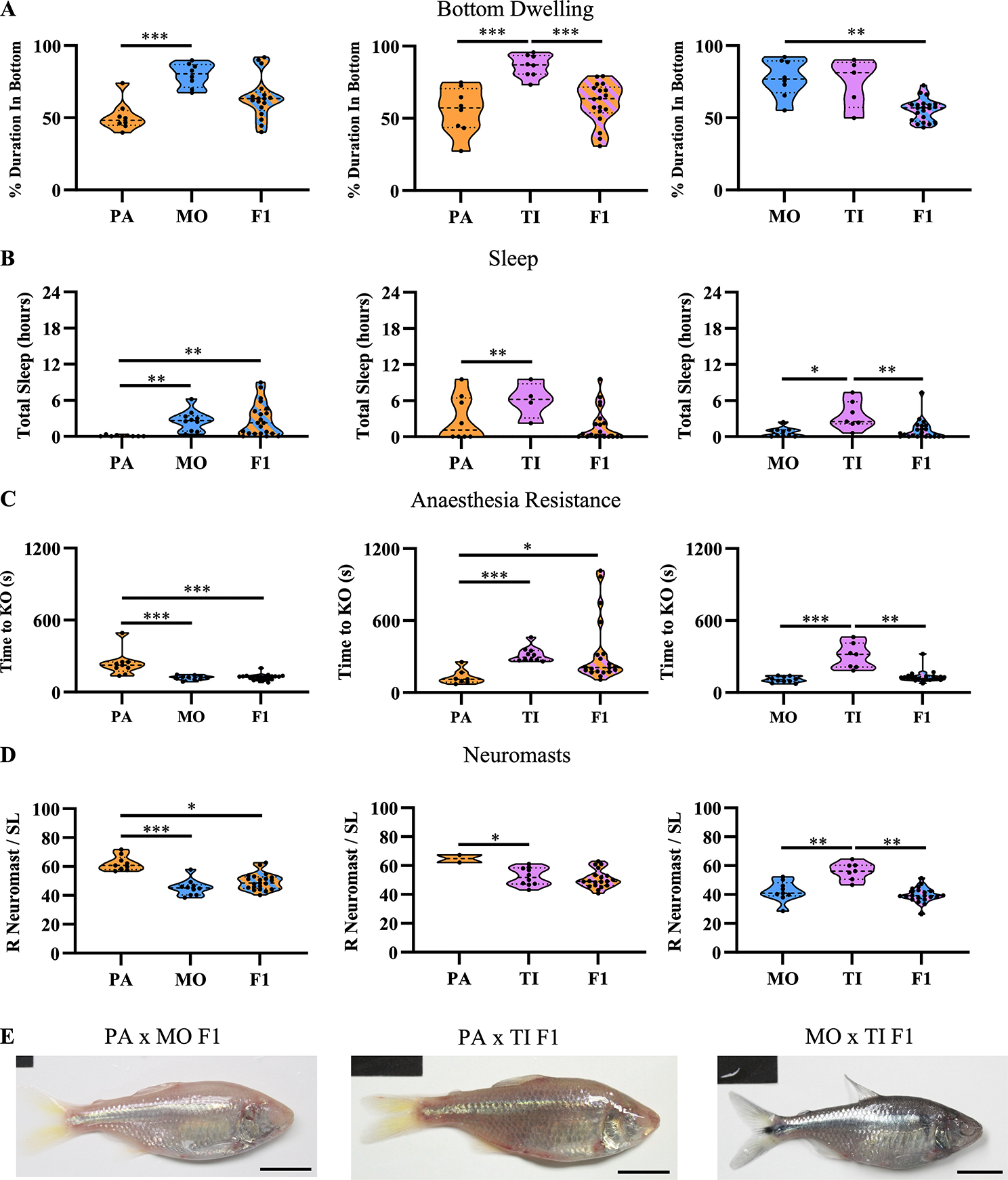
Analysis of cave/cave F1 hybrids suggests that multiple traits show non-complementation. (A) Percent time spent bottom dwelling during the novel tank assay in cave and cave–cave F1 hybrid populations (*N*: PA = 8, MO = 8, F1 = 17; PA = 8, TI = 8, F1 = 18; MO = 8, TI = 5, F1 = 21). (B) Total sleep duration over 23.5 h in cave and cave–cave F1 hybrid populations (*N*: PA = 9, MO = 10, F1 = 21; PA = 8, TI = 4, F1 = 20; MO = 10, TI = 7, F1 = 22). (C) Time spent responsive following the addition of tricaine anaesthetic in cave and cave–cave F1 hybrid populations (*N*: PA = 9, MO = 10, F1 = 21; PA = 7, TI = 10, F1 = 20; MO = 10, TI = 7, F1 = 22). KO = knock out. (D) Number of superficial neuromasts over the right suborbital 3 bone, corrected by standard length (SL) (*N*: PA = 9, MO = 10, F1 = 21; PA = 2, MO = 10, F1 = 19; MO = 9, TI = 7, F1 = 21). Right-side neuromasts over standard length were graphed, but statistical comparisons are of right-side neuromast residuals, calculated as described in §2. (E) Full-body images of PA × MO F1s, PA × TI F1s and MO × TI F1s. Scale bars = 1 cm. Data points indicate values of phenotypes of individual fish. Exact *p* values are reported in electronic supplementary material, table S1. PA, MO and TI represent Pachón, Molino and Tinaja, respectively. Levels of significance are represented with asterisks: *p* < 0.05 = *, *p* < 0.01 = **, *p* < 0.001 = ***, *p* < 0.0001 = ****.

To further examine whether these traits may have evolved through similar or different underlying genetic bases, we phenotyped cave–cave F1 hybrids for a subset of traits to assess for complementation. None of the cave–cave hybrids spent more time bottom-dwelling than fish from individual cave populations (figure 3A). While Pachón–Molino F1 hybrids sleep significantly more than Pachón cavefish, these hybrids spent a similar amount of time sleeping to Molino cavefish, and no increases to more surface-like levels of sleep were observed in cave–cave hybrid fish compared with both cavefish from populations from which they derived (figure 3B). We also did not observe evidence for a shift to reduced anaesthesia resistance (figure 3C) or reduced neuromast number (figure 3D, electronic supplementary material, figure S4) in cave–cave hybrids relative to both cavefish populations from which they were derived. Similar to previous reports of non-complementation between Molino and Pachón cavefish for albinism [33], our Pachón–Molino hybrid fish were albino (figure 3E). Both Pachón–Tinaja and Molino–Tinaja F1 hybrids are pigmented, which is consistent with the lack of albinism in Tinaja cavefish and the recessive nature of albinism in both Molino and Pachón populations. However, Pachón–Tinaja and Molino–Tinaja F1 hybrids qualitatively differ in pigmentation (figure 3E). While albinism (the presence or absence of melanin pigmentation) in *A. mexicanus* is monogenic [26,33], prior work has demonstrated that the amount of pigmentation is a quantitative trait and that different cave–cave F1 crosses differ in their amounts of pigmentation [27,28,60]. Our qualitative findings are consistent with this. All cave–cave F1 hybrids had no external eyes as adults (figure 3E). Prior research has demonstrated that cave–cave hybrids have larger eyes during larval stages and have larger, skin-covered internal eyes than either cave parent during adult stages [60,65,66]. We were unable to quantify eye size in our cave–cave crosses because they are not visible externally (figure 3E).

Together, these data show that, with the exception of pigmentation in Molino–Tinaja F1 hybrids, shifts to surface-like phenotypes were not observed for the traits assessed in cave–cave hybrid fish. This lack of robust complementation in cave–cave hybrid fish suggests that at least a subset of the same loci may underlie the evolution of these traits across these populations. Together with the patterns of inheritance found in cave–surface crosses and some quantitative differences in traits between cave populations, these results suggest that both shared and distinct genetic underpinnings contribute to the repeated evolution of traits across these three cavefish populations.

### (d) Genetic correlations are observed across cavefish populations

**Figure 4.**
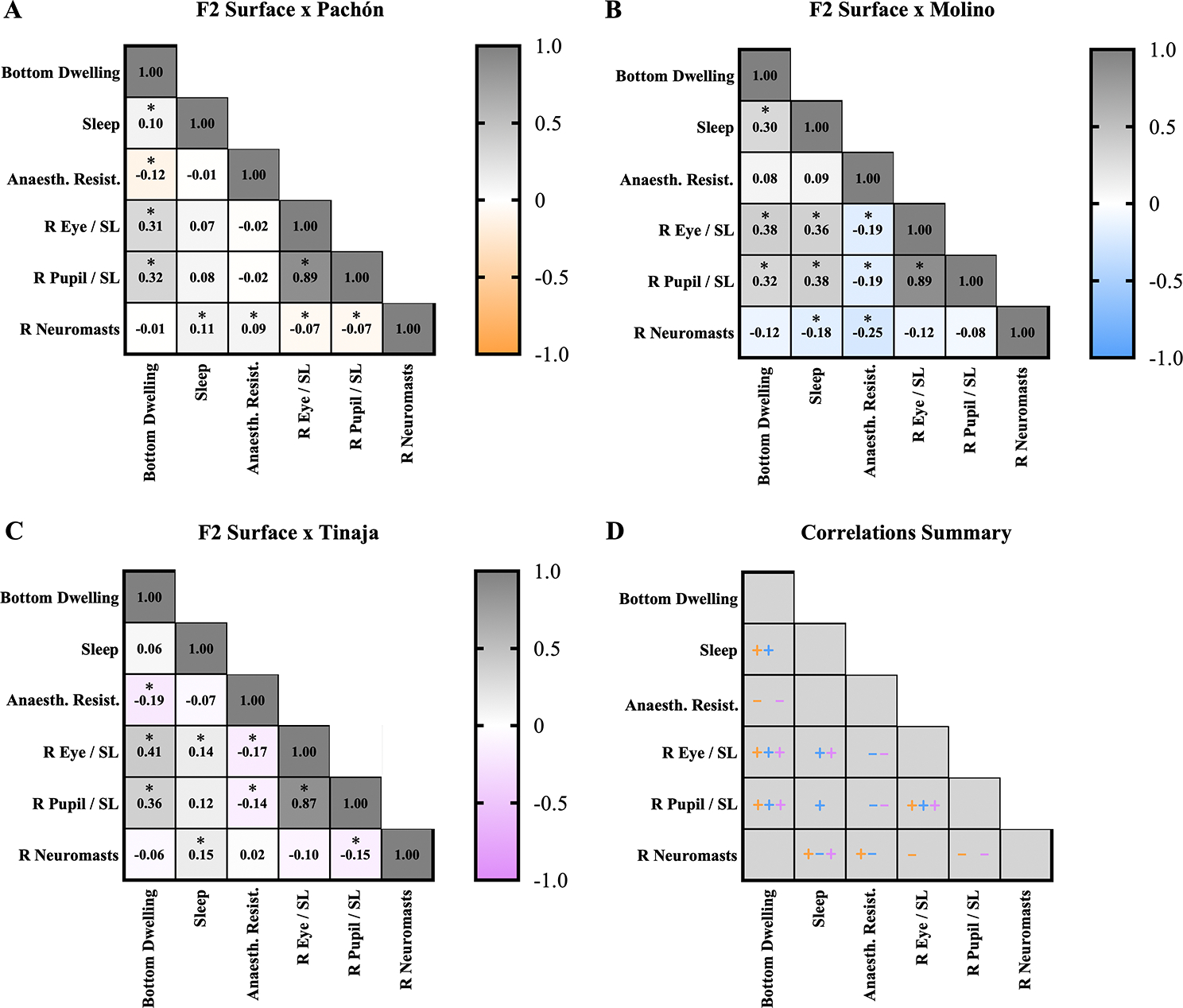
Repeatedly occurring trait covariances in three F2 populations suggest potential shared genetic mechanisms for divergent traits. Matrices of Spearman’s rank correlations of traits measured in (A) F2 Pachón hybrids, (B) F2 Molino hybrids, and (C) F2 Tinaja hybrids. (D) A matrix summary of statistically significant correlations and the directionality of those correlations across all three cave F2 populations. Colour of symbols indicates the population. Matrices report the Spearman’s *r* value. Statistically significant covariances are marked with a black asterisk. Exact *p* values and sample sizes are reported in electronic supplementary material, table S1. SL, standard length.

Next, we investigated whether there was evidence indicating a role of shared genetic loci underlying the evolution of multiple traits within cave populations. Analysis of correlations between traits in cave–surface F2 hybrid fish provides a powerful opportunity to investigate whether shared genetic architecture underlies different traits. Previous work in *A. mexicanus* found that multiple traits in the surface–Pachón F2 hybrids covary with one another and have overlapping QTL peaks in the genome [36,38,39,43]. However, whether cave-derived traits are correlated in other populations of surface–cave hybrids is largely unknown. To investigate this, we compiled pairwise Spearman correlation matrices for traits in each of the three surface–cave F2 hybrid populations. Within each cave population, we identified multiple correlated traits, suggesting that multiple traits evolved together owing to linkage or pleiotropy (figure 4). These correlations were typically weak or moderate, with the exception of eye and pupil size, which were strongly correlated in all three populations (figure 4), suggesting that shared genetic loci between pairs of traits do not explain all of the genetic architecture underlying these traits within populations.

Next, we assessed whether the same traits were correlated across populations. In all three populations, bottom dwelling and eye size, bottom dwelling and pupil size, and eye size and pupil size were all positively correlated (figure 4). Neuromasts and sleep significantly correlated in all populations, but with different directionalities (figure 4D). These traits were negatively correlated in surface–Molino hybrids but positively correlated in surface–Pachón and surface–Tinaja hybrids, potentially reflecting the Pachón’s and Tinaja’s shared lineage (figure 4). Multiple correlations were observed between two populations. In surface–Pachón and surface–Tinaja F2 hybrids, bottom dwelling and anaesthesia resistance were negatively correlated, as were pupil size and neuromast number (figure 4A,C,D). In surface–Molino and surface–Tinaja F2 hybrids, eye size and sleep were positively correlated, and eye size and anaesthesia resistance were negatively correlated, as were pupil size and anaesthesia resistance (figure 4B–D). In surface–Pachón and surface–Molino F2 hybrids, bottom dwelling and sleep positively correlated. Several correlations were identified in only one population. In surface–Pachón F2 hybrids, neuromasts and eye size were positively correlated (figure 4A). In surface–Molino F2 hybrids, sleep and pupil size were positively correlated (figure 4B). Interestingly, nine of the eleven trait correlations observed in two or more populations were in the same direction.

**Figure 5.**
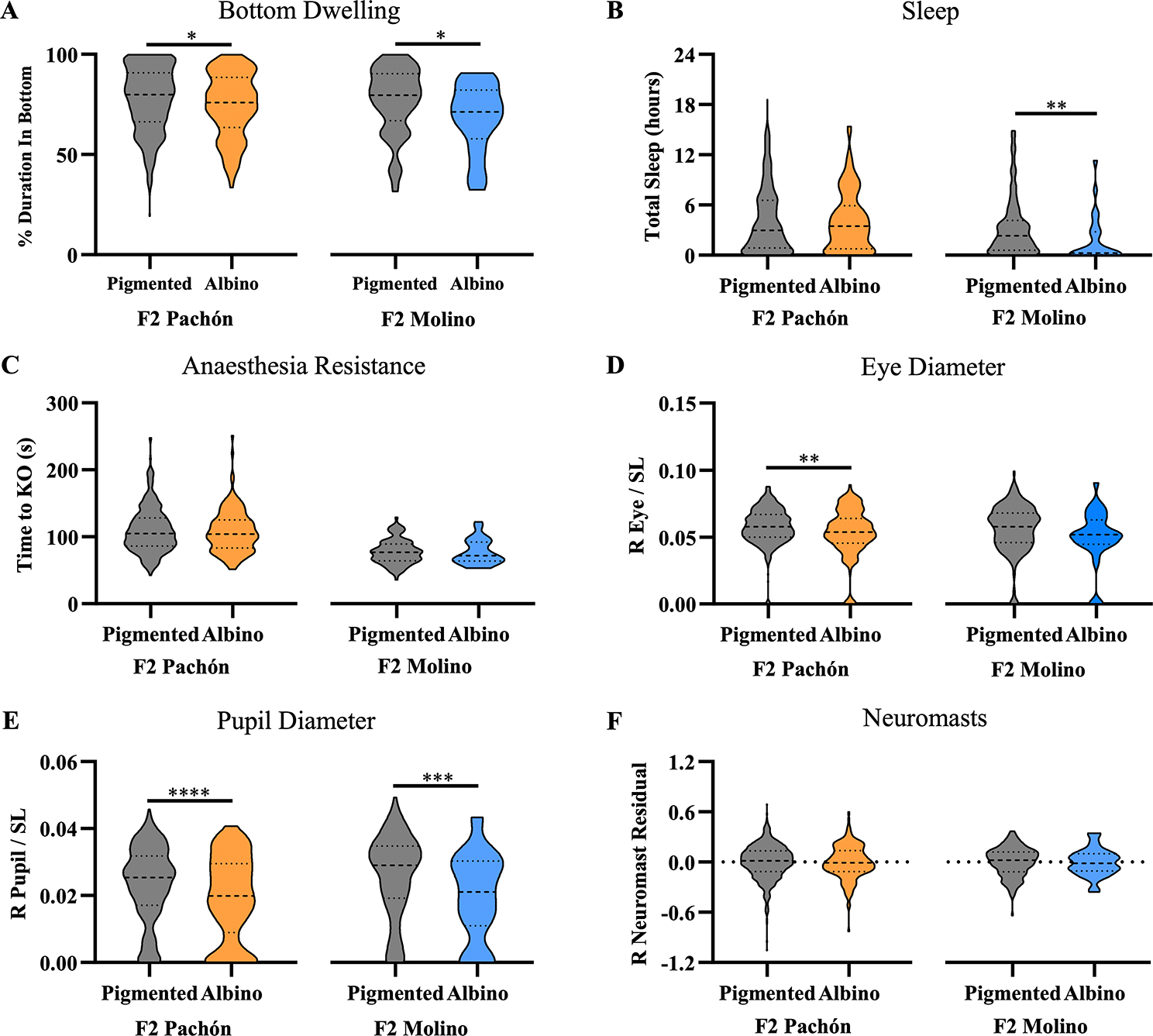
Several traits repeatedly differ between pigmented and albino F2s in Pachón and Molino hybrid populations. (A) Percent time spent bottom dwelling in the novel tank assay (*N*: F2 PA pigmented = 551, albino = 136; F2 MO pigmented = 117, albino = 25). (B) Total sleep over 23.5 h during day 0 (*N*: F2 PA pigmented = 391, albino = 97; F2 MO pigmented = 124, albino = 25). (C) Time spent responsive following the addition of tricaine anaesthetic (*N*: F2 PA pigmented = 554, albino = 139; F2 MO pigmented = 96, albino = 26). (D) Dorsal–ventral right eye diameter corrected by standard length (SL) (N: F2 PA pigmented = 611, albino = 166; F2 MO pigmented = 151, albino = 33). (E) Dorsal–ventral right pupil diameter corrected by standard length (*N*: F2 PA pigmented = 610, albino = 165; F2 MO pigmented = 151, albino = 33). (F) Residuals of F2 neuromasts located over the right suborbital 3 bone (*N*: F2 PA pigmented = 608, albino = 164; F2 MO pigmented = 149, albino = 32). Exact *p* values are reported in electronic supplementary material, table S1. Levels of significance are represented with asterisks: *p* < 0.05 = *, *p* < 0.01 = **, *p* < 0.001 = ***, *p* < 0.0001 = ****.

Finally, we also assessed our surface–Pachón and surface–Molino F2 hybrids for albinism and investigated whether there were differences in traits between pigmented and albino F2s. Anaesthesia resistance and neuromasts were not different between pigmented and albino F2s in either cave population (figure 5C,F, electronic supplementary material, figure S5C–E). However, albino F2 hybrids derived from both Pachón and Molino populations had smaller pupils and eyes than their pigmented siblings (figure 5D,E, electronic supplementary material, figure S5A,B). Additionally, both albino Pachón and Molino F2 hybrids exhibited reduced bottom dwelling (figure 5A). Albino Molino F2 hybrids also exhibited reduced sleep relative to pigmented hybrids, although no difference was observed in Pachón F2 hybrids (figure 5B). Together, these data suggest that shared inheritance between different traits, whether owing to linkage or pleiotropy, may repeatedly occur across morphological and behavioural traits.

### (e) Directly testing for a role of pleiotropy in observed trait covariances via *oca2*-mutant surface fish

**Figure 6.**
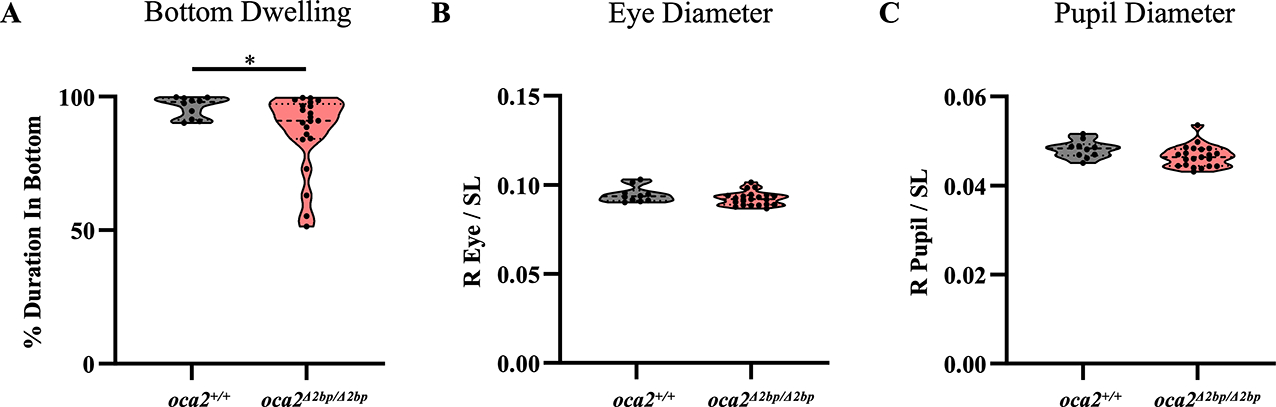
Albino surface fish spend less time bottom dwelling than wild-type surface fish. (A) Percent time spent bottom dwelling in the novel tank assay between wild-type and *oca2*^*Δ2bp/Δ2bp*^ surface fish (*N*: *oca2*^+/+^ = 10, *oca2*^*Δ2bp/Δ2bp*^ = 21). (B) Dorsal–ventral right eye diameter corrected by standard length (SL) between wild-type and *oca2*^*Δ2bp/Δ2bp*^ surface fish (*N*: *oca2*^+/+^ = 10, *oca2*^*Δ2bp/Δ2bp*^ = 21). (C) Dorsal–ventral right pupil diameter corrected by standard length between wild-type and *oca2*^*Δ2bp/Δ2bp*^ surface fish (*N*: oca2^+/+^ = 10, *oca2*^*Δ2bp/Δ2bp*^ = 21). Data points indicate values for phenotypes of individual fish. Exact *p* values are reported in electronic supplementary material, table S1. Levels of significance are represented with asterisks *p* < 0.05 = *, *p* < 0.01 = **, *p* < 0.001 = ***, *p* < 0.0001 = ****.

Traits linked to albinism present a powerful opportunity to directly test whether pleiotropy or linkage impacts evolution of traits because the causative genetic locus for albinism in cavefish, the *oca2* gene, is known [33,56]. To determine whether *oca2* impacts traits that differed between albino and pigmented hybrids, i.e. bottom dwelling behaviour and eye morphology, we utilized a line of surface fish that had CRISPR-Cas9-induced mutations in *oca2* which, when homozygous, result in albinism (see electronic supplementary material, figure S6C, [56]). We compared bottom dwelling and eye size in wild-type (*oca2*^*+/+*^) and albino (*oca2*^*Δ2bp/Δ2bp*^) surface fish siblings. Albino surface fish had reduced bottom dwelling behaviour relative to their wild-type siblings (figure 6A) but they did not have any significant differences in eye size (figure 6B, electronic supplementary material, figure S6A) or pupil size (figure 6C, electronic supplementary material, figure S6B), consistent with previous work showing that mutating *oca2* in surface fish larvae is not sufficient to alter eye size [42]. Bottom dwelling behaviour in *A. mexicanus* is probably a vision-independent trait [32], suggesting that this is probably not owing to *oca2-*based visual processing differences. The results from a Hedge’s *G* test indicate that mutations in *oca2* probably have a large effect size on bottom dwelling behaviour in surface fish (*G* = 0.7484, electronic supplementary material, table S1). These data suggest that genetic variation at the *oca2* locus may play a role in reducing bottom dwelling behaviour in albino cavefish populations.

## 4. Discussion

Repeated phenotypic evolution in response to similar environments provides a powerful opportunity to assess the extent that suites of traits evolve through shared genetic mechanisms both within and between populations. Through quantifying a variety of traits in surface fish, cavefish, hybrid crosses and *oca2* mutant surface fish, we demonstrate that many cave-derived traits are correlated, suggesting that shared genetic architecture, via pleiotropy or close linkage, has repeatedly contributed to the evolution of the cave phenotype.

### (a) Shared inheritance and concentrated genetic architectures

Cave animals across taxa have repeatedly evolved suites of similar traits, including reductions in the visual system, loss or reduction of pigmentation and enhancement of non-visual sensory systems [67]. Similarly, multiple morphological, physiological and behavioural traits have evolved repeatedly in *A. mexicanus* cavefish populations [34,35,68]. Thus, cave animals provide a powerful system in which to study multi-trait evolution. Across multiple independent cavefish populations, we identified correlations among traits in cave–surface F2 hybrids, which may be consistent with a role for concentrated genetic architectures underlying multiple traits involved in cave adaptation. This is in line with previous QTL mapping research that has found that *A. mexicanus* cavefish have concentrated QTL regions [36–39]. Our results extend earlier work showing correlations for distinct traits in Pachón hybrids [36,37,39]. These prior works also identified overlapping QTL for multiple traits, and future QTL mapping of our dataset will give insight into the commonality of overlapping QTL in populations outside of Pachón.

### (b) Pleiotropy, linkage and the case study of *oca2*

Determining whether co-variation between traits is owing to pleiotropy can be challenging, as differentiating between pleiotropy and close linkage is difficult [69]. Indeed, multi-trait evolution through concentrated genetic loci has been attributed to pleiotropy and to linkage in other species. For example, a repeatedly identified mechanism underlying multi-trait evolution is evolution of traits via supergenes—chromosomal inversions that contain multiple genes, and within which multiple individual genes impact different traits [20]. Supergenes have been associated with multi-trait evolution across species, including redpoll finches, butterflies, and deer mice [70–72]. Conversely, multiple instances of pleiotropic genes contributing to the evolution of traits have been identified. One of these is the *foraging* gene in *Drosophila melanogaster,* which was initially identified as playing a role in larval foraging behaviour, and has since been implicated in natural variation in other traits, including learning and memory, sleep and temperature tolerance [73].

The genes and genetic changes that contribute to the evolution of traits in cave *A. mexicanus* are largely unknown, however, the basis of albinism is known. Albinism in at least two populations of *A. mexicanus* cavefish is owing to deletions in *oca2* [33,56]. This provides the opportunity to investigate traits covarying with albinism to differentiate between pleiotropy and close linkage. Here, we performed a direct test of the role of *oca2* in multi-trait evolution. In addition to the role that *oca2* plays in albinism in cavefish [33,56,74], *oca2* has been implicated in catecholamine levels, anaesthesia resistance, larval feeding behaviour and sleep loss [42,44–46]. We found that albino hybrids differed in their stress response, sleep and eye/pupil size relative to pigmented siblings. Examination of two of these traits in *oca2* mutant surface fish confirmed that *oca2* impacts bottom dwelling, but not eye or pupil size when mutated in surface *A. mexicanus*. Together, this is consistent with a pleiotropic role of *oca2* contributing to the evolution of reduced stress response behaviours in cavefish.

Prior research in mice and humans has demonstrated that *oca2* impacts visual acuity and muscular control of the eye [75,76], but, to our knowledge, *oca2* has not been implicated in any eye size differences. In *A. mexicanus* cavefish, in addition to defects in the lens that play a large role in eye degeneration [77], alterations to the retinal pigmented epithelium (RPE) contribute to eye degeneration [78]. While we did not see an effect of *oca2* on eye size here, as *oca2* is expressed in the RPE, including in *A. mexicanus* [79], we cannot rule out the possibility that *oca2* impacts the morphology of the eye, either through changes in morphology not observable through our methods of measurement or through changes in eye size that were too small to detect. Moreover, we cannot rule out the possibility that when mutated alone in surface fish, *oca2* may not be sufficient to alter eye size, but in combination with other genetic changes, mutations in *oca2* impact eye and/or pupil size in cavefish. Thus, while our data are consistent with differences in eye size between albino and pigmented cave–surface hybrid fish being owing to linkage rather than pleiotropy, further studies are needed to fully elucidate the impact of loss of *oca2* on cavefish eye development.

### (c) Repeated evolution and alternative evolutionary paths

Across all three cave–surface F2 hybrid populations, we find both shared and population-specific correlations among traits, suggesting that some aspects of cave evolution may be shaped by common genetic architectures while others have probably evolved through distinct, lineage-specific correlations. Across all three populations, we found correlations between eye phenotypes and bottom dwelling, similar to the direction of trait change seen in cavefish evolution. Several trait correlations were present only in Pachón and Tinaja, and several additional trait correlations were observed between Molino and Tinaja as well. We also observed population-specific correlations, particularly in Molino hybrids. These results suggest that while some multi-trait correlations are common across populations, others have evolved only in specific cave populations, indicating that both shared and lineage-specific genetic architectures underlie repeated evolution. Whether the same or distinct loci underlie trait correlations observed across different cave populations remains an open question. Both crosses and genetic mapping studies suggest that for many traits in cavefish, both shared and distinct genetic loci contribute to the repeated evolution of these traits [33,40,66]. Genetic mapping studies of these traits in multiple cave populations may provide additional insight into whether repeated instances of co-variation across cave populations are owing to the same or different genomic regions.

Notably, many of the observed correlations among traits align with the directions of phenotypic evolution that we repeatedly observe in cavefish, suggesting that the genetic variance–covariance matrix is at least partly structured along axes that facilitate the direction of phenotypic evolution that we observe in the caves [80]. One caveat is that while phenotypic correlations in F2s provide insight into the potential shared genetic bases, shared environmental conditions within the laboratory environment, measurement issues, or an unmeasured latent variable may influence the phenotypic correlations we observe here.

In conclusion, our findings suggest that in *A. mexicanus,* concentrated genetic architectures underlie multiple cave-adapted traits, owing either to pleiotropy or close linkage. Moreover, we find that multi-trait evolution via these concentrated genetic architectures occurs across different cave populations. Through functional validation, we find that *oca2* influences bottom dwelling and is probably closely linked with other loci that influence eye size and pupil size. Future studies that integrate QTL mapping and further functional validation can help identify cases of pleiotropy versus linkage and shed additional light on the genetic basis of repeated evolution.

## Supplementary Material

macros

Suppl Figures and Methods

STable 1

STable 2

Supplementary material is available online [81].

Electronic supplementary material is available online at https://doi.org/10.6084/m9.figshare.c.8351250.

## Data Availability

Data and code are available as supplementary material.
