# Supplementary material for: Analysis of multi-trait evolution across independently evolved cavefish populations reveals shared and independent evolution of suites of traits
Source: Proc Biol Sci. Author manuscript; Available in PMC 2026 Jun 25. (PMC13296793; doi:10.1098/rspb.2025.2719)
Supplement: macros [file NIHMS2175969-supplement-macros.zip › Macros for Publication/README.docx]

Each macro and its function:

**convert_092025_mse.sh** - Cygwin macro used to convert Ethovision files from utf16 to utf8 for the sleep macro

**sleepMacro_250816_editAnalysisRange_counts-fix.py** - Anaconda macro used to take utf8 data from Ethovision and then exports an excel spreadsheet with sleep data

**Neuromast_macro_05052025_pixel_edit.py** - Neuromast macro used to count neuromasts via background subtraction
