# Supplementary material for: Analysis of multi-trait evolution across independently evolved cavefish populations reveals shared and independent evolution of suites of traits
Source: Proc Biol Sci. Author manuscript; Available in PMC 2026 Jun 25. (PMC13296793; doi:10.1098/rspb.2025.2719)
Supplement: Suppl Figures and Methods [file NIHMS2175969-supplement-Suppl_Figures_and_Methods.pdf]

Supplemental Figures and Methods for:

Stefan Choy, Maya Enriquez, Aubrey E. Manning, Rianna Ambosie, Briley F. Mullin, Roberto Rodriguez-Morales, Jennah Abdelaziz, Sarah Jacobson, Evan Lloyd, Naresh Padmanaban, Alli Kimmel, Solomia Lapko, Isabel Carino-Bazan, Helena Bilandžija, Alex C. Keene, Erik Duboue, Suzanne McGaugh, Johanna E. Kowalko. Analysis of multi-trait evolution across independently evolved cavefish population reveals shared and independent evolution of suites of traits. Proceedings of the Royal Society B.

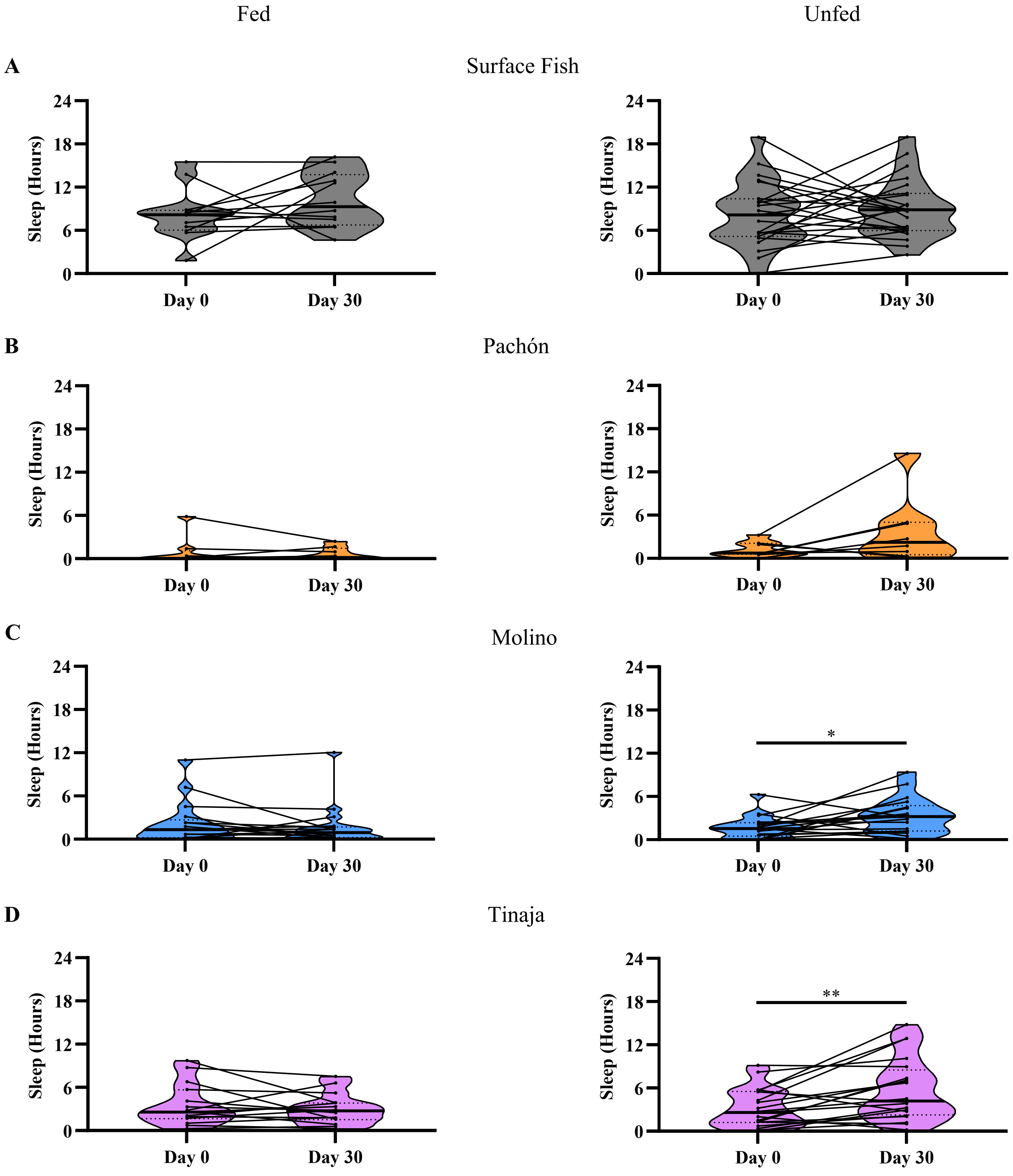

Supplemental Figure 1. Change in sleep over 30 days in Surface, Pachón, Molino, and Tinaja populations. A) Change in total sleep in fed (N = 12) and unfed (N = 23) surface fish. B) Change in sleep in fed (N = 8) and unfed (N = 8) Pachón fish. C) Change in sleep in fed (N = 17) and unfed (N = 18) Molino fish. D) Change in sleep in fed (N = 15) and unfed (N = 20) Tinaja fish. The fish present in these graphs were from all experiments performed for this assay. Data points indicate phenotypes of individual

fish. Exact p values are reported on Supplemental Table 1. Levels of significance are represented with asterisks: $P > 0.05$ = *, $P > 0.01$ = **, $P > 0.001$ = ****, $P < 0.0001$ = ****.

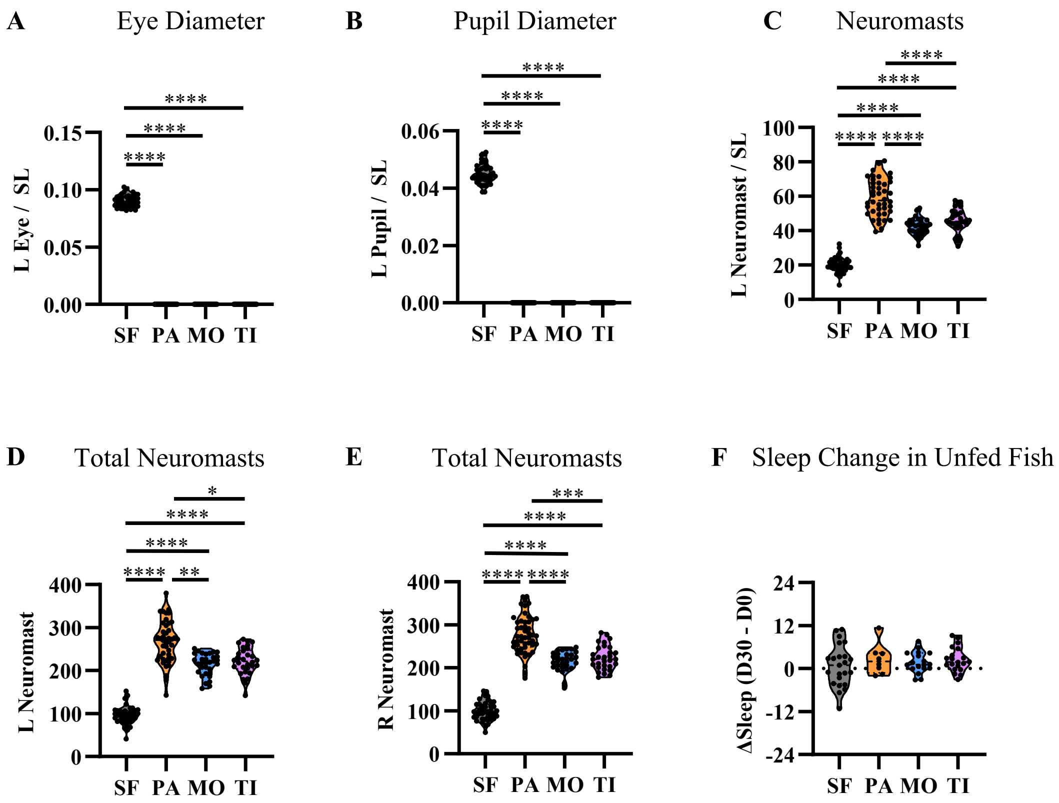

Supplemental Figure 2. Left-sided morphological data across *A. mexicanus* populations. A) Dorsal-ventral measurements of the left eye diameter, corrected by standard length. (N: SF = 43, PA = 49, MO = 33, TI = 30). B) Dorsal-ventral measurements of the left pupil diameter, corrected by standard length (N: SF = 43, PA = 49, MO = 33, TI = 30). C) Number of superficial neuromasts over the left suborbital 3 bone, corrected by standard length (N: SF = 41, PA = 42, MO = 32, TI = 30). Left-side neuromasts over standard length were graphed, but statistical comparisons are of left-side neuromast residuals, calculated as described in the Methods. D) Total number of superficial neuromasts over the left suborbital bone length (N: SF = 41, PA = 44, MO = 32, TI = 30). E) Total number of superficial neuromasts over the right suborbital 3 bone (N: SF = 41, PA = 49, MO = 32, TI = 30). F) Change in sleep over 30 days of starvation in surface, Pachón, Molino, and Tinaja fish (N: SF = 24, PA = 16, MO = 29, TI = 26). Exact p values and sample sizes are reported on Supplemental Table 1. Data points indicate values for phenotypes of individual fish. SF, PA, MO, and TI represent surface fish, Pachón, Molino, and Tinaja, respectively. Levels of significance are represented with asterisks $p < 0.05$ = *, $p < 0.01$ = **, $p < 0.001$ = ***, $p < 0.0001$ = ****.

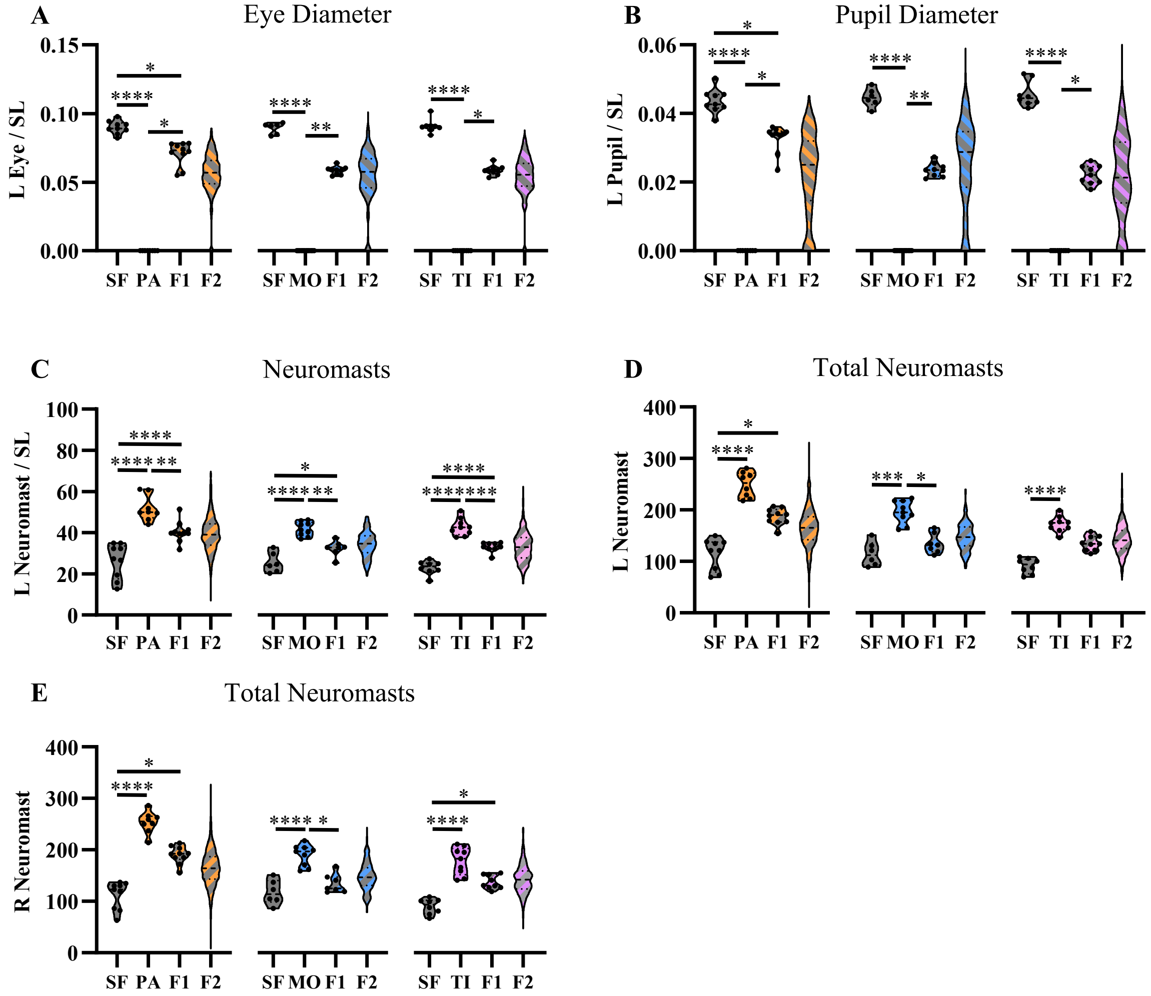

Supplemental Figure 3. Left-sided morphological data in parental, F1 hybrid, and F2 hybrid populations. A) Dorsal-ventral measurements of the left eye diameter, corrected by standard length (N: SF = 9, PA = 8, F1 = 10; SF = 6, MO = 12, F1 = 9; SF = 8, TI = 9, F1 = 9). B) Dorsal-ventral measurements of the left pupil diameter, corrected by standard length (N: SF = 9, PA = 8, F1 = 10; SF = 6, MO = 12, F1 = 9; SF = 8, TI = 9, F1 = 9). C) Number of superficial neuromasts over the left suborbital 3 bone, corrected by standard length (N: SF = 9, PA = 8, F1 = 10; SF = 6, MO = 9, F1 = 7; SF = 8, TI = 9, F1 = 9). Left-side neuromasts over standard length were graphed, but statistical comparisons are of left-side neuromast residuals, calculated as described in the Methods. D) Total number of superficial neuromasts over the left suborbital 3 bone (N: SF = 9, PA = 8, F1 = 10; SF = 6, MO = 9, F1 = 7; SF = 8, TI = 9, F1 = 9). E) Total number of superficial neuromasts over the right suborbital 3 bone (N: SF = 9, PA = 8, F1 = 10; SF = 6, MO = 9, F1 = 7; SF = 8, TI = 9, F1 = 9). Statistics were performed only on surface, cave, and F1 hybrid populations. Data points indicate values of phenotypes of individual fish. Data points were not shown for F2 fish. Exact p values are reported on Supplemental Table 1. SF, PA, MO, and TI represent surface fish, Pachón, Molino, and Tinaja, respectively. Levels of significance are represented with asterisks $p < 0.05$ = *, $p < 0.01$ = **, $p < 0.001$ = ***, $p < 0.0001$ = ****.

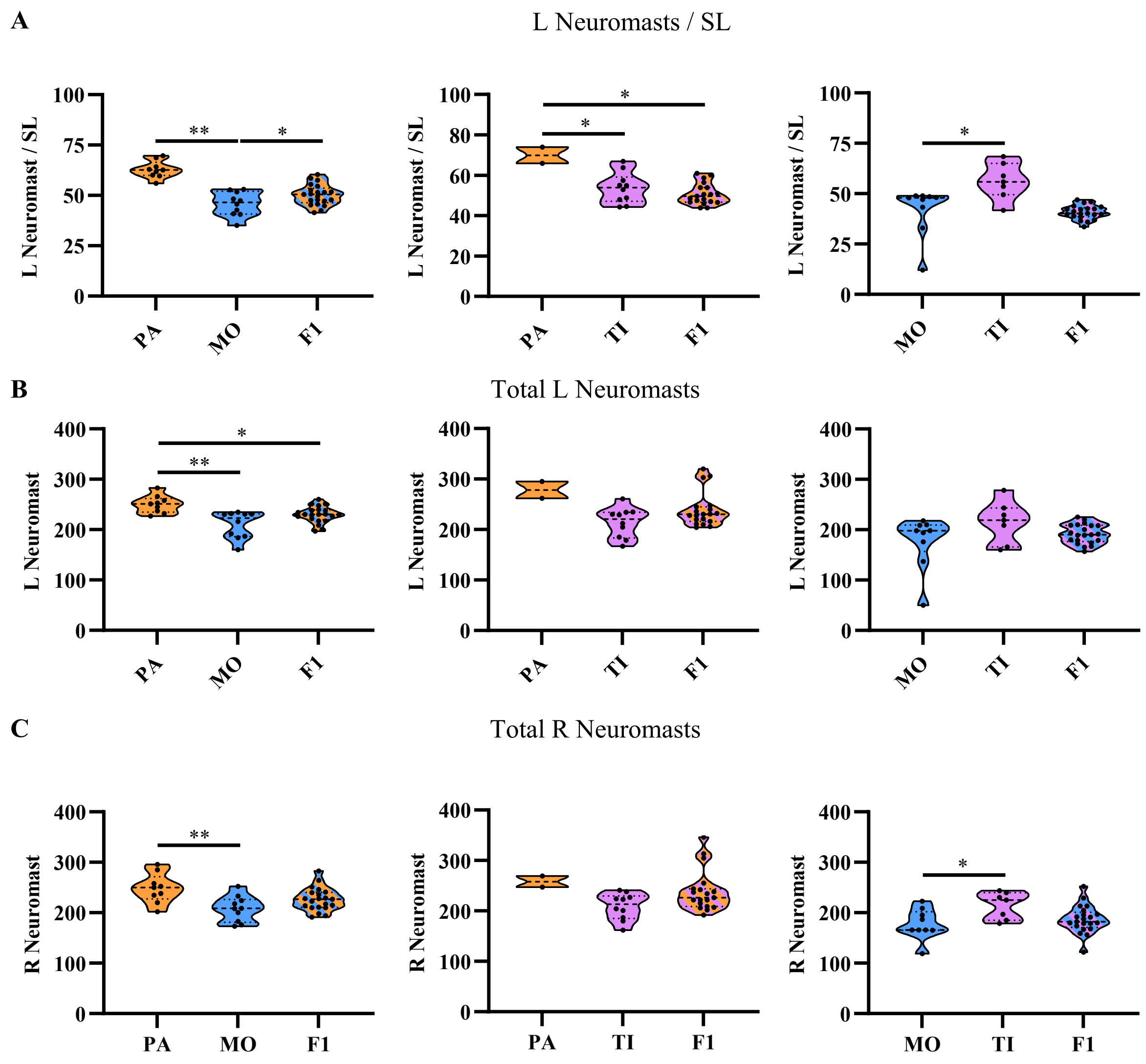

Supplemental Figure 4. Left-sided neuromasts in cave and F1 cave/cave hybrid populations. A) Number of superficial neuromasts over the left suborbital 3 bone, corrected by standard length (N: PA = 9, MO = 10, F1 = 21; PA = 2, TI = 10, F1 = 19; MO = 9, TI = 7, F1 = 21). Left-side neuromasts over standard length were graphed, but statistical comparisons are of left-side neuromast residuals, calculated as described in the Methods. B) Total number of superficial neuromasts over the left suborbital 3 bone (N: PA = 9, MO = 10, F1 = 21; PA = 2, TI = 10, F1 = 19; MO = 9, TI = 7, F1 = 21). C) Total number of superficial neuromasts over the right suborbital 3 bone (N: PA = 9, MO = 10, F1 = 21; PA = 2, TI = 10, F1 = 19; MO = 9, TI = 7, F1 = 21). Data points indicate values of phenotypes of individual fish. Data points were not shown for F2 fish. Exact p values are reported on Supplemental Table 1. PA, MO, and TI represent Pachón, Molino, and Tinaja, respectively. Levels of significance are represented with asterisks p < 0.05 = *, p < 0.01 = **, p < 0.001 = ***, p < 0.0001 = ****.

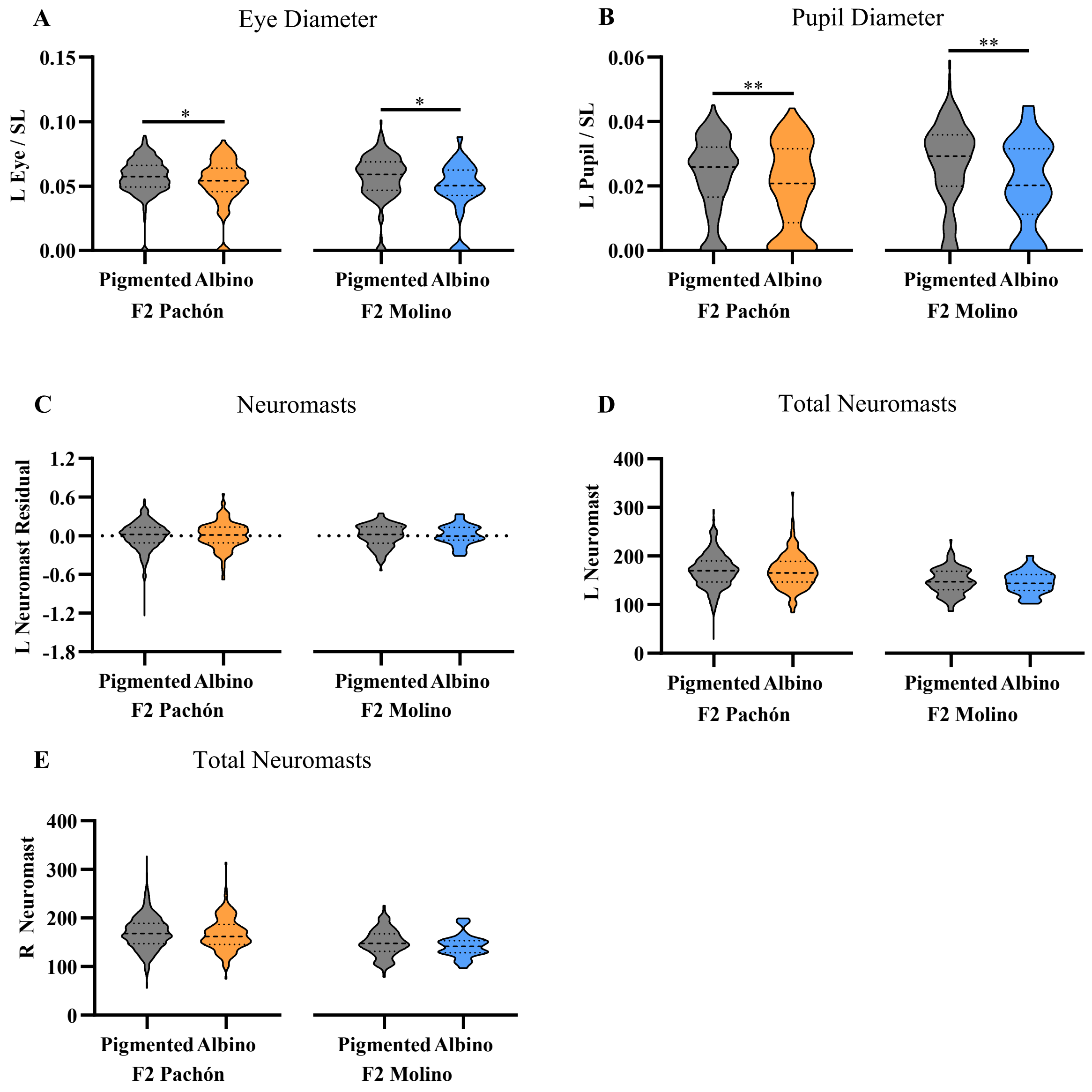

Supplemental Figure 5. Left-sided morphological traits in F2 Pachón and F2 Molino hybrids, split by pigmentation. A) Dorsal-ventral measurements of left eye diameter, corrected by standard length (N: F2 PA Pigmented = 610, Albino = 163; F2 MO pigmented = 148, albino = 33). B) Dorsal-ventral measurements of the left pupil diameter, corrected by standard length (N: F2 PA pigmented = 609, albino = 162; F2 MO pigmented = 148, albino = 33). C) Residuals of F2 neuromasts located over the left suborbital 3 bone (N: F2 PA pigmented = 604, albino = 162; F2 MO pigmented = 145, albino = 31). D) Total number of superficial neuromasts over the left suborbital 3 bone (N: F2 PA pigmented = 605, albino = 162; F2 MO pigmented = 145, albino = 31). E) Total number of superficial neuromasts over the right suborbital 3 bone (N: F2 PA pigmented = 608, albino = 164; F2 MO pigmented = 149, albino = 32). Exact p values are reported on Supplemental Table 1. Levels of significance are represented with asterisks $p < 0.05$ = *, $p < 0.01$ = **, $p < 0.001$ = ***, $p < 0.0001$ = ****.

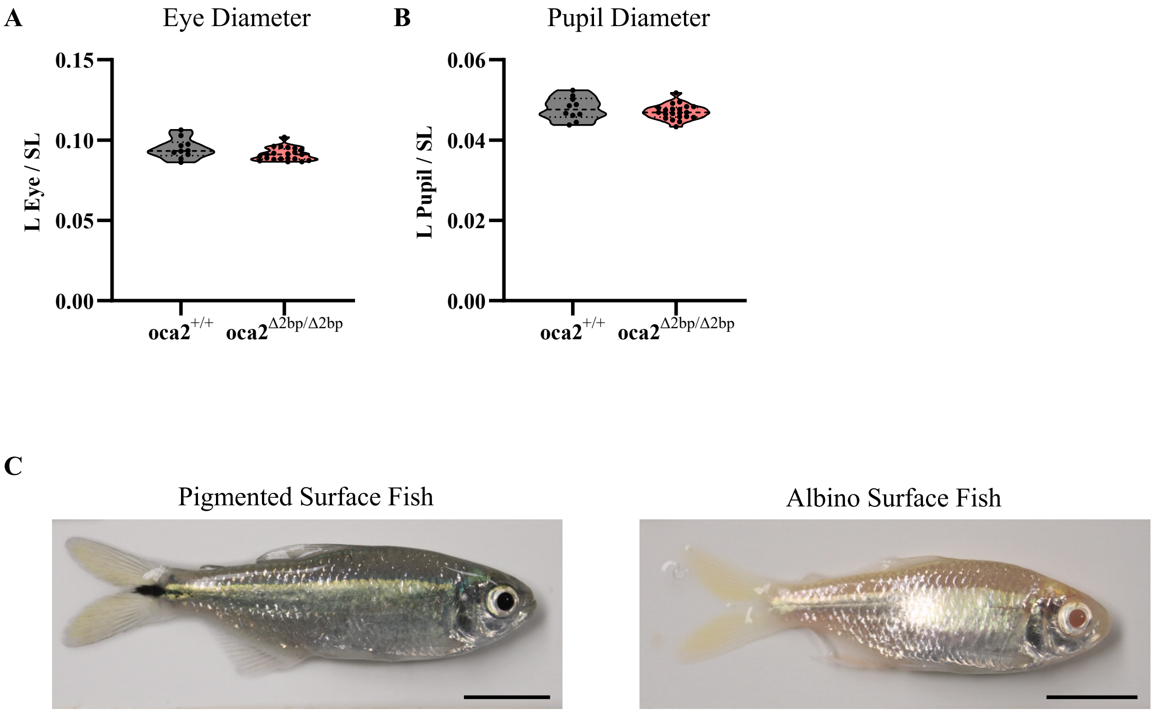

Supplemental Figure 6. Eye morphology in wild-type and *oca2* mutant surface fish. A) Dorsal-ventral measurements of left eye diameter, corrected by standard length (N: $oca2^{+/+}$ = 10, $oca2^{\Delta 2bp/\Delta 2bp}$ = 21). B) Dorsal-ventral measurements of the left pupil diameter, corrected by standard length (N: $oca2^{+/+}$ = 10, $oca2^{\Delta 2bp/\Delta 2bp}$ = 21). C) Full-body images of pigmented and albino surface fish siblings. Scale bar = 1cm. Exact p values are reported on Supplemental Table 1. Data points indicate values of phenotypes of individual fish. Levels of significance are represented with asterisks $p < 0.05$ = *, $p < 0.01$ = **, $p < 0.001$ = ***, $p < 0.0001$ = ****.

Supplemental Methods

Novel tank

Bottom-dwelling in a novel tank was assessed as a measure of stress, as previously described [1]. Fish were removed from their home tank and placed in a 500ml beaker to acclimate for 10 minutes. The water and fish were then gently transferred to a new tank (21.3cm x 12.7cm x12.7cm) filled with 2 liters of water. Warm white LED strip lights were used to illuminate the novel tank setup. The 10 minutes following fish placement into the assay tank were recorded at 30 frames per second (fps) with a Basler ace acA1300-60gm GigE Mono camera (Edmund Optics: 88327) with Basler PylonViewer software. The novel tank videos were analyzed via Ethovision (version 17.0.1630), an automatic tracking software. In Ethovision, the analysis arena was bisected into top and bottom zones. Time spent in the bottom zone was converted to a percent of the total and reported. Videos for which the entire 10-minute recording did not complete, or for which Ethovision was unable to track the fish for more than 10% of the total video were excluded from further analysis. A subset of novel tank videos were analyzed using a machine learning approach, which combines pose-estimation and automatic behavioral annotation programs to infer bottom dwelling behavior [2]. Any novel tank assay that had more than a 15% difference between Ethovision and the machine learning approach was re-analyzed using Ethovision as another quality control step. Additionally, any fish with less than 30% bottom dwelling was re-analyzed.

Sleep and food restriction

Following the novel tank assay, fish were moved into experimental sleep tanks (45 x 17 x 17cm) [3]. Tanks were divided into five segments using opaque dividers and one fish was placed into each of the first four segment, while the fifth segment housed a sponge bubbler. All assays were backlit with 850nm infrared strip lights and recorded with a modified Microsoft LifeCams (Amazon: B004ABO7QI) fitted with an IR-pass filter, so that illumination was constant between light and dark conditions. Overhead warm white LED lights were set on a 14:10 L/D light cycle. Fish were allowed to acclimate for four days and were tested on the fifth day of being in the tank for initial sleep phenotyping. Fish were fed bloodworms once per day during acclimation. Fish were then deprived of food for 30 days, during which water changes with conditioned water were performed every 5 days. After the 30 day no rations period, fish spent another 7 days in the segmented tanks and were fed bloodworms once per day. A subset of parental populations (surface fish, Pachón, Molino, and Tinaja) were fed bloodworms once per day to serve as controls.

The recorded sleep videos were processed using Ethovision behavior tracking software to quantify the amount of time spent sleeping based on velocity measurements [4,5]. The utf-16 encoded data files were then converted into utf-8 files and processed via a custom python code (see Supplemental Files) to extract the amount of time spent sleeping. Specifically, fish found to be moving at less than 4 cm/s for more than 60 or more seconds were considered to be asleep, as in [4]. Only 23.5 hours were analyzed, with analysis starting 30 minutes after lights on (zeitgeber time 0.5). Here, we report the total hours of sleep across the 23.5 hours.

Anesthesia resistance

Response to anesthesia was measured after at least seven days of recovery from food restriction, as previously described [6]. Fish were placed into a 500mL beaker filled with 200mL conditioned water and allowed to acclimate for at least 10 minutes. Following acclimation, 200mL of 200mg/L MS-222, or tricaine, conditioned water solution, adjusted to proper pH with sodium bicarbonate, was added to the beaker, and the time to unconsciousness was recorded to the nearest second. Unconsciousness was defined as the fish no longer responding to prodding with a plastic pipette to the head and operculum. Following these trials, fish were immediately returned to their home tank and allowed to recover.

Morphology

Fish were stained with a 28.9mg/L DASPEI solution for 60 minutes in the dark to prevent breakdown of DASPEI, as previously described [7]. Following a brief treatment with chilled tricaine, photos of the lateral view of the head and fluorescently labelled neuromasts were taken on a fluorescent dissecting microscope (Leica M165 FC). Additionally, full-body photos were taken with a Canon Rebel T7 camera (Amazon: B07C2Z21X5) with a centimeter ruler and color standard in frame for standard length quantification. Standard length was measured from the tip of the snout to the base of the tail using FIJI [8].

Eye analysis

Eye and pupil sizes were quantified by measuring the dorsal-ventral diameter of the eye and pupil using FIJI [8]. A subset of eyes or pupils from F2 hybrid fish were not fully visible (obscured by skin, occluded by orbital bone, etc.) and were not quantified, as the edges of the structure were not identifiable. Both eye and pupil size were correlated with fish length and had an isometric relationship to length (see 'Statistics' section), thus, both metrics are reported as corrected for standard length by dividing by standard length.

Neuromast analysis

Superficial neuromasts within the boundary of the third suborbital bone (SO3) were quantified on each side of the fish via a custom ImageJ code [9] (see Supplemental Materials). The quantification of neuromasts was limited to the SO3 area since neuromasts in this section of the animal have previously been shown to be directly correlated with food finding abilities and sleep [5,10]. The code has users outline the SO3 anatomical area and then utilizes a background subtraction algorithm to count neuromasts within this boundary. Each image was then manually checked and corrected to ensure accurate neuromast quantification. Any images that exhibited signs of DASPEI overstaining or fish that had anatomical abnormalities that significantly warped the SO3 area were excluded from analysis. The anatomical right standardized neuromasts were used in this analysis, with the left side being reported in the supplemental figures. As the number of neuromasts correlated with the size of the fish, we corrected neuromast number for this trait (see Statistics section). However, we also reported values for total neuromast number in the supplemental figures and data.

Genotyping oca2 fish

Briefly, fin clips were digested in 100uL 50mM NaOH at 95ºC for 30 minutes, and 10uL 1M Tris-HCl pH 8.0 was added after as a buffer. Genotyping utilized allele-specific forward primers; 5′-CTGGTCATGTGGGTCTCAGC-3 binds to the wild-type allele and 5′-TCTGGTCATGTGGGTCTCATT-3′ binds to the mutant allele. The same reverse primer, 5′-TTTCCAAAGATCACATATCTTGAC-3′, was used for both reactions. The PCR annealing temperature was set to 58ºC, and gel electrophoresis was performed to visualize amplicons, as previously described [11,12].

Statistical Analysis

For all morphological traits (eye size, pupil size, neuromast number), to determine if standardizing with length was a sufficient size correction, we ran a global test comparing models with and without population-by-length interactions. Our analysis indicated that while pupil size and eye size had an isometric relationship to length, neuromast number was allometric, and we proceeded to run a regression analysis on neuromasts only. This examination in neuromasts did not support population-specific differences in slopes, indicating a shared allometric relationship. Based on this result, we applied a

regression-based size correction using the shared-slope model (log(neuromast) ~ log(length) + population), and computed the log observed-to predicted ration (equivalent to the residual from the log-log regression). These size corrections were used to conduct post-hoc Tukey-adjusted pairwise population comparisons based on estimated marginal means evaluated at a common reference body length. In addition to the population-specific comparisons of length, for our analysis of pigmented and albino individuals in F2 hybrid populations, we used the shared slope model (log(neuromast) ~ log(length) + pigmentation) in surface x Pachon F2 and surface x Molino F2 individuals, with the same post-hoc Tukey adjusted pairwise comparisons as described above. For F2s of each population, we made a linear model for log-transformed right neuromasts compared to the average length within the population. We then calculated the estimated marginal means (residuals) by calculating the difference between the predicted number of neuromasts based on this model and the actual number of neuromasts observed. These statistics can be found in the length-standardized graph captions.

1. Chin JSR, Gassant CE, Amaral PM, Lloyd E, Stahl BA, Jaggard JB, Keene AC, Duboue ER. 2018 Convergence on reduced stress behavior in the Mexican blind cavefish. *Dev. Biol.* **441**, 319–327.
2. Padmanaban N, Ambosie R, Choy S, Marcus S, Nilsson SRO, Keene AC, Kowalko JE, Duboué ER. 2025 Automated behavioral profiling using neural networks reveals differences in stress-like behavior between cave and surface-dwelling Astyanax mexicanus. *J. Exp. Zool. B Mol. Dev. Evol.* **344**, 352–362.
3. Worsham M, Fernandes VFL, Settle A, Balaan C, Lactaoen K, Tuttle LJ, Iwashita M, Yoshizawa M. 2019 Behavioral tracking and neuromast imaging of Mexican cavefish. *J. Vis. Exp.* (doi:10.3791/59099)
4. Yoshizawa M, Robinson BG, Duboué ER, Masek P, Jaggard JB, O'Quin KE, Borowsky RL, Jeffery WR, Keene AC. 2015 Distinct genetic architecture underlies the emergence of sleep loss and prey-seeking behavior in the Mexican cavefish. *BMC Biol.* **13**, 15.
5. Jaggard J, Robinson BG, Stahl BA, Oh I, Masek P, Yoshizawa M, Keene AC. 2017 The lateral line confers evolutionarily derived sleep loss in the Mexican cavefish. *J. Exp. Biol.* **220**, 284–293.
6. Bilandžija H, Abraham L, Ma L, Renner KJ, Jeffery WR. 2018 Behavioural changes controlled by catecholaminergic systems explain recurrent loss of pigmentation in cavefish. *Proc. Biol. Sci.* **285**. (doi:10.1098/rspb.2018.0243)
7. Yoshizawa M, Goricki S, Soares D, Jeffery WR. 2010 Evolution of a behavioral shift mediated by superficial neuromasts helps cavefish find food in darkness. *Curr. Biol.* **20**, 1631–1636.
8. Schindelin J *et al.* 2012 Fiji: an open-source platform for biological-image analysis. *Nat. Methods* **9**, 676–682.
9. McGaugh SE *et al.* 2019 Evidence for rapid phenotypic and behavioural shifts in a recently established cavefish population. *Biol. J. Linn. Soc. Lond.* **129**, 143–161.
10. Yoshizawa M, O'Quin KE, Jeffery WR. 2013 QTL clustering as a mechanism for rapid multi-trait evolution. *Commun. Integr. Biol.* **6**, e24548.
11. O'Gorman M *et al.* 2021 Pleiotropic function of the oca2 gene underlies the evolution of sleep loss and albinism in cavefish. *Curr. Biol.* **31**, 3694-3701.e4.
12. Choy S *et al.* 2025 Mutations in the albinism gene oca2 alter vision-dependent prey capture behavior in the Mexican tetra. *J. Exp. Biol.* **228**, jeb249881.
